# Advanced CMR Techniques in Anderson-Fabry Disease: State of the Art

**DOI:** 10.3390/diagnostics13152598

**Published:** 2023-08-04

**Authors:** Andrea Ponsiglione, Marco De Giorgi, Raffaele Ascione, Carmela Nappi, Luca Sanduzzi, Antonio Pisani, Serena Dell’Aversana, Alberto Cuocolo, Massimo Imbriaco

**Affiliations:** 1 Department of Advanced Biomedical Sciences, University of Naples Federico II, 80131 Naples, Italy; a.ponsiglionemd@gmail.com (A.P.); marcodeg9@gmail.com (M.D.G.); c.nappi@unina.it (C.N.); luca.sanduzzi1@gmail.com (L.S.); cuocolo@unina.it (A.C.); 2 Department of Diagnostic Imaging, Pineta Grande Hospital, 81030 Castel Volturno, Italy; raffoascio@gmail.com; 3 Department of Public Health, University of Naples Federico II, 80131 Naples, Italy; antonio.pisani13@gmail.com; 4 Department of Radiology, Santa Maria delle Grazie Hospital, ASL Napoli 2 Nord, 80078 Pozzuoli, Italy; dellaversanaserena@gmail.com

**Keywords:** cardiac magnetic resonance, Anderson-Fabry disease, strain, T1 mapping, T2 mapping, perfusion imaging, hybrid imaging

## Abstract

Anderson-Fabry disease (AFD) is a rare multisystem X-linked lysosomal storage disorder caused by α-galactosidase A enzyme deficiency. Long-term cardiac involvement in AFD results in left ventricular hypertrophy and myocardial fibrosis, inducing several complications, mainly arrhythmias, valvular dysfunction, and coronary artery disease. Cardiac magnetic resonance (CMR) represents the predominant noninvasive imaging modality for the assessment of cardiac involvement in the AFD, being able to comprehensively assess cardiac regional anatomy, ventricular function as well as to provide tissue characterization. This review aims to explore the role of the most advanced CMR techniques, such as myocardial strain, T1 and T2 mapping, perfusion and hybrid imaging, as diagnostic and prognostic biomarkers.

## 1. Introduction

**Figure 1:**
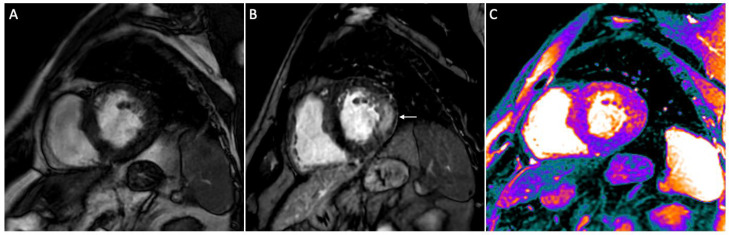
Short-axis CMR images of a 56-year-old male patient with AFD. Cine image shows left ventricle hypertrophy (**A**), while on post contrast image a typical mid-wall area of late gadolinium enhancement (LGE) is present within the infero-lateral segment (arrow in (**B**)). On native T1 mapping (**C**) a diffuse decrease of native myocardial T1 values (purple areas) is found (global native T1: 945 ms), apart from the infero-lateral wall.

Anderson-Fabry disease (AFD) is a lysosomal storage disorder caused by a mutation of the gene encoding Galactosidase α (GLA) located on X chromosome (Xq22.1), resulting in α-galactosidase A enzyme deficiency [1] and lowered enzymatic activity levels to less than 25–30% of the mean normal value [2]. This results in an accumulation of glycosphingolipids, mainly globotriaosylceramide, in different types of cells, with subsequent organ/tissue damage of the cardiovascular, renal, gastrointestinal, cerebrovascular, neurological, auditory, ocular, and cutaneous systems [3]. The clinical phenotype of the disease is affected by the severity and the variability of the age of onset and can be severe and precocious in the classic forms of AFD or mild and late in the variant forms [4]. Females may be symptomatic despite X-linked transmission, but their manifestations are generally less severe than those of males [5]. Cardiac involvement may occur in up to 70% of patients [4] and represents the most common cause of death in these patients. Cardiac damage includes left ventricular hypertrophy (LVH), myocardial inflammation and fibrosis, secondary to abnormal glycosphingolipids deposition in myocytes [6], which can present with heart rhythm disturbances, coronary artery disease and heart failure [5]. An early diagnosis of organ damage and subsequent early treatment are essential for an optimal management of the patient, allowing to prevent major complications and slowing down the progression of the disease [7]. The cardiac manifestations of AFD often match other cardiomyopathies, with diagnosis being challenging only based on clinical evaluation [8]. The role of cardiac imaging is mainly expressed with echocardiography and cardiovascular magnetic resonance (CMR) [9]. In particular, CMR represents the predominant noninvasive and multi-parametric imaging modality for the assessment of cardiac involvement in the AFD, being able to comprehensively assess cardiac regional anatomy, ventricular function as well as to provide tissue characterization [9]. The most common morphological finding is represented by LVH, with the contribution of the papillary muscle not to be underestimated [10]. Another characteristic finding is the presence of substitution areas of fibrosis identifiable with late gadolinium enhancement (LGE) sequences (Figure 1) [11]. Commonly LGE affects the basal inferolateral wall of the left ventricle and has been described in about 50% of affected patients, although the reason for this tropism is not yet clear [12].

According to a recent expert consensus document, in the absence of contraindications, CMR should be considered in all adult patients to assess cardiac anatomy and function, as well as the presence of myocardial fibrosis at initial evaluation (class IIa of recommendation) [13]. Additionally, CMR may be considered every 5 years in adult patients to assess the progression of fibrosis and LV function depending on disease severity (class IIb of recommendation).

The aim of our review is to highlight the most advanced CMR techniques currently available for cardiac AFD assessment with a look at hybrid positron emission tomography (PET)/MR imaging, underlining their value in the early diagnosis and prognostic implications.

## 2. Strain

Strain represents the deformation generated by the application of a force, with myocardial strain consisting of the percent change in myocardial length from a relaxed to a contractile status. With a similar work scheme, myocardial strain can be assessed both by echocardiographic images and CMR by monitoring a fixed spatial coordinate and material particles passing through, following specific analysis algorithms then modeled for each imaging modality [14]. The assessment relies on the global and regional study of different spatial components of the contractile function of the longitudinal strain (LS), circumferential strain (CS) or radial strain (RS) directions [15]. CMR myocardial feature tracking (FT) is a new validated 2D imaging technique that can be applied to standard cine sequences and allows to measure the myocardial deformation with a precise measurement of both global and regional myocardial strain [16]. It has aroused interest because it guarantees the measurement of myocardial strain without the need to specified acquisition and complex post-processing [17]. This method may be helpful to distinguish between myocardium deformations in different heart diseases including hypertrophic cardiomyopathy, cardiac amyloidosis and myocarditis [18]. It has been widely accepted in the modern literature that strain analysis can implement information of the classical global and segmental functional analysis, going beyond the limits of the traditional parameters used to describe LV function as the ejection fraction (EF) [19]. In particular, strain alterations have been described with preserved EF [20], representing sensitive marker of sub-clinical myocardial dysfunction with independent prognostic significance across several cardiovascular diseases [20,21]. Furthermore, CMR-FT enables an easier measurement of all cardiac chambers, including the relatively thin-walled atria as well as the right ventricle, which may be hardly assessed with myocardial tagging [22].

In a study of 20 AFD patients and 20 healthy controls, Zhao et al. [23] showed that myocardial strain can identify myocardial deformation in AFD at different stages, being able to monitor disease severity. Notably, LV global longitudinal strain (GLS) was significantly reduced in all three AFD groups (LVH/LGE negative; LVH+/LGE either + or −; LGE+/LV wall thinning/heart failure) (all *p* < 0.05) compared to healthy controls. Vijapurapu et al. [24] performed a prospective study of 221 AFD patients and 77 healthy volunteers (HV) who underwent CMR, ECG, and blood biomarkers. In this study, they concluded that GLS in AFD is associated with an increased indexed LV mass (LVMi), storage, and the presence of ECG alterations. In early disease, when LVH is not present yet, impaired GLS is instead linked to a reduction in native T1, suggesting that mechanical dysfunction is earlier than evidence of sphingolipid deposition (low T1). Halfmann et al. [25] supported, with a study of 58 patients with proven AFD and HV, the importance of assessing the left atrium (LA) deformation patterns as an integral part of the diagnostic work-up in AFD. In detail, the Authors calculated LA strains, manually tracing endocardial and epicardial borders in a single slice at end-diastole in the 2-,3-, and 4-chamber-views and showed how LA reservoir strain could distinguish between early-phase AFD patients and HV. In addition, atrial strain analysis has been proven effective in the diagnosis of early AFD. This study also showed a significant correlation between LA strains and disease severity as measured by T1 and LVMi [25].

An overview of the main studies on strain with CMR is presented in Table 1.

## 3. T1 Mapping

T1 mapping is a relatively recent established and reproducible CMR technique aimed at characterizing myocardial tissue [26]. In particular, native T1 mapping estimates the intrinsic longitudinal relaxation time of the myocardium tissue without administration of a contrast agent [27]. Assessment of myocardial T1 can be performed using sequences that require one short breath-hold per slice, with native T1 being higher in fibrosis, edema and amyloid accumulation and lower in iron overload and focal fat infiltration [28]. The quantitative T1 signal from the myocardium is analyzed pixel-by-pixel and post-processed to derive a colored map of the myocardium in which the different T1 values are represented by different colors [29]. Therefore, native T1 mapping with CMR may be useful to assess early cardiac involvement of AFD and distinguish this latter from other causes of LVH [30]. A lowering of the native T1 values can be given by the overload of glycosphingolipids in the initial stages of the disease, anticipating the subsequent development of LVH and myocardial dysfunction; however, the influence of the demographic characteristics of the patients and the imaging acquisition protocols used must be considered [31]. Of note, to distinguish iron overload and focal fat infiltration, both of which exhibit low myocardial T1 values, quantifying T2* can be instrumental. Specifically, a T2* value greater than 20 msec indicates the absence of cardiac iron overload [32].

Ponsiglione et al. [31] recently conducted a meta-analysis of 982 subjects which confirmed the lowering of native myocardial T1 values in AFD patients compared to that of healthy controls, pointing out that the degree of T1 shortening in AFD is influenced by gender and LVH. Overall, the weighted mean native T1 values was 984  ±  47 ms in AFD patients while that of healthy controls was 1016  ±  26 ms (*p*  <  0.0001). In particular, the higher myocardial native T1 values observed in pre-menopausal women may be in part explained by the thinner myocardial walls with a predisposition to partial-volume effect as well as by the different myocardial characteristics due to the hormone status and lower hematocrit values [33]. In addition, since AFD gen0etic inheritance is recessive X-linked, in heterozygous women enzyme α-galactosidase A activity is partially maintained by the presence of wild-type gene on the other allele, resulting in mild to absent accumulation of lipids into myocardial cells and possibly higher values of T1 mapping compared to the male population. Moreover, long-standing cardiac AFD leads to a hypertrophic phenotype, mainly with concentric LVH [31]. Therefore, myocardial native T1 values progressively become lower as LV wall thickness increases. Late hypertrophic stages of AFD are characterized by the development of interstitial fibrosis, with focal pseudo-normalization of native T1 times. Ponsiglione et al. [31] also revealed the need to standardize the method considering the variability of values obtainable using different protocols and depending on different vendors.

Camporeale et al. [34] conducted a study on 44 patients with genetically confirmed AFD and without LVH using a Shortened Modified Looked Locker inversion recovery sequence and measuring T1 values in the mid-septum, anterior, inferior, and lateral LV walls. In AFD global cohort, T1 values showed a significant correlation with LV mass, LV wall thickness, Sokolow lyon index (an ECG measurement used to assess LVH), Mainz severity score index (a scoring system used to assess disease severity and progression in AFD patients) and LA volume. The Authors demonstrated that the presence of low T1 values is a risk factor for disease worsening at 12-month follow-up, thus representing a potential new marker in prognostic stratification and therapeutic approach [34]. In cases of AFD with LVH, native myocardial T1 is substantially lower compared to other causes of LVH and T1 lowering is an earlier marker of cardiac involvement than other parameters of CMR as confirmed by Sado et al. [29].

T1 mapping abnormalities were also found in AFD-positive patients without evidence of concurrent LVH and Pica et al. demonstrated that the reduction in T1 before the onset of LVH is associated with early diastolic and systolic changes as measured by echocardiography [35]. Thompson et al. claim that low myocardial T1 values without contrast is the most sensitive and specific CMR parameter in patients with AFD, irrespective of gender and LV morphology and function [36].

In a recent study conducted by Osborne et al., the performance of a prognostic model for predicting adverse cardiac outcomes in patients with AFD was evaluated [37]. Notably, the highest-performing, internally validated, multivariable model comprised age, native myocardial T1 dispersion (standard deviation of per voxel myocardial T1 relaxation times) and LVMi as key predictors.

Furthermore, T1 mapping has been also assessed to the effect of migalastat on cardiac involvement in 16 AFD patients [38]. The Authors demonstrated that following an 18-month treatment with migalastat, there was a stabilization of LV mass, along with a promising trend towards improved exercise tolerance and a tendency towards an increase in T1 values, while extracellular volume (ECV) remained unchanged.

A study from another research group found an overall stable course of CMR-measured LV mass and function in AFD patients treated with migalastat, but no clear trend was found for T1 values [39].

Since in AFD interstitial spaces are reduced by the intracellular accumulation of sphingolipids, ECV is normal or even decreased, and its role is limited to more advanced stages of disease, when fibrosis occurs [36,40].

An overview of the main studies focused on T1 mapping with CMR is presented in Table 2.

## 4. T2 Mapping

Previous papers have demonstrated that T2-weighted (T2w) CMR and in particular prolonged myocardial T2 relaxation time can be observed in patients with AFD, mainly secondary to the biophysical and biochemical characteristics of the tissue [41,42]. In particular, Imbriaco et al. have shown that myocardial water as well as lipid alterations may lead to an abnormal prolongation of the myocardial T2 relaxation time in patients with AFD and to an overall increase in signal intensity compared to that of hypertrophic patients and HV [41]. In addition, in patients with AFD undergoing enzyme replacement therapy (ERT) CMR documented a significant reduction in myocardial T2 relaxation time, a significant decrease in maximal myocardial thickness and in total LV mass, highlighting the value of myocardial T2 relaxation time to monitor treatment effects [42]. T2w CMR imaging is also commonly used for the assessment of myocardial inflammation because myocardial T2 increases in the presence of edema [43]. However, the T2w assessment is mainly a qualitative approach and is prone to several limiting factors in its clinical adoption as the quality of the image obtained, the reproducibility and the subjective evaluation of the finding. Regional myocardial T2 mapping aims to reduce these limitations with a quantitative approach by directly quantifying local myocardial inflammation and edema [44]. T2 values directly reflect free water content of the tissue of interest, enabling to quantify edema. T2 mapping sequences generate parametric images that are based on the transverse relaxation time for each voxel for a T2 decay curve of a series of T2w images. The image obtained is analyzed visually on a grey (or color) scale and T2 can be rapidly quantified within regions of interest, with T2 relaxation time being longer in case of edema [45]. Augusto et al. [46] conducted a prospective international multicenter study of 186 AFD patients, 58 with other cardiovascular disease (28 patients with hypertrophic cardiomyopathy, 30 with chronic myocardial infarction) and 59 HV. The Authors showed that in subjects with LGE T2 values were regionally and globally significantly higher compared to patients without LGE. Interestingly, LGE T2 elevation was higher in AFD compared to chronic myocardial infarction or hypertrophic cardiomyopathy. Finally, a high basal inferolateral wall T2 was linked to both electrocardiographic alterations and global longitudinal strain impairment. Nordin et al. [47] compared 20 patients starting ERT with 18 patients with early disease and 18 with advanced disease over 1 year at 3 different centers. Over 1 year, patients with advanced disease showed a significant increase in T2 in the LGE area (*p* = 0.023) and troponin (*p* = 0.036), while there was no significant difference in maximum wall thickness and LVMi. Additionally, the Authors speculated that chronic ERT use does not fully normalize LVH, T1, T2, or troponin levels. Furthermore, the disease appeared to progress slowly in both early untreated and more advanced treated stages, but in distinct ways. In early disease, there was more storage and the development of LVH. In contrast, advanced disease showed increasing inflammation and progressively impaired strain. Finally, the Authors concluded that newly treated subjects displayed a clear signal of the ERT effect, with a small reduction in LV mass and normalization of T1 once LVH is present.

An overview of the main studies focused on T2 mapping is presented in Table 3.

## 5. CMR Perfusion Imaging

AFD may cause endothelial storage and microvascular dysfunction and several papers have shown the value of PET as the gold standard modality in this setting, although it is limited by the lower spatial resolution and the use of ionizing radiation [48,49,50]. Quantitative perfusion CMR has been developed generating perfusion maps similar to T1 or T2 maps, providing good and reproducible results compared to the published PET data [51]. In particular, Knott et al. conducted a prospective observational study in 44 AFD patients with LVH and 27 HV with multi-parametric CMR including vasodilator stress perfusion mapping for assessment of myocardial blood flow (MBF). The results of this study show that patients with AFD and increased LVH, sphingolipid deposition and myocardial fibrosis had lower myocardial perfusion compared to HV. However, stress MBF was also lower in LVH-negative AFD compared with controls, thus this reduction in stress MBF might occur early during the course of the disease, even before the onset of myocardial damage. Structural changes in the myocardial microvasculature of patients with AFD have been also explored using biopsy. In particular, Chimenti et al. [52] compared endo-myocardial biopsies of 13 AFD patients with angina to a control cohort of AFD patients without chest pain. Although the endothelial cells were swollen due to glycosphingolipids accumulation, arteriolar luminal narrowing was present and linked to hypertrophy and hyperplasia of the smooth muscle cells and increased fibrosis of the intimal and medial layers. Furthermore, in patients without angina there was less luminal narrowing. It is therefore possible that abnormalities in microvascular perfusion environment, as assessed by multiparametric CMR, could precede myocyte storage and myocardial dysfunction.

## 6. Hybrid PET/MR

The study of traditional imaging, focused on the evaluation of density and water content, may be implemented using specific biomarkers that interact with the surrounding environment. The observed molecular changes can produce meaningful images, opening new avenues for the detection of early myocardial damage and for diseases characterization. In particular, 18F-fluorodeoxyglucose positron emission tomography (18F-FDG PET) is a highly sensitive non-invasive molecular imaging technique for metabolically active processes that utilize glucose such as an energy source [53]. The sensitivity of the cardiac 18F-FDG PET to identify hypermetabolic glucose-avid with 18F-FDG uptake, allows to implement the anatomical and myocardial structure information of the CMR [54].

However, the primary constraint of 18F-FDG PET is linked to the physiological myocardial glucose metabolism [55]. In regular circumstances, a healthy myocardium utilizes a blend of free fatty acids and glucose for energy. Consequently, distinguishing between myocardial inflammation and normal physiological myocardial activity necessitates the inhibition of physiological myocardial uptake. This can be accomplished by shifting the myocardial metabolism from glucose consumption to the utilization of free fatty acids [56].

Nappi et al. [56] first demonstrated that integrated 18F-FDG PET/MR can be considered for the early diagnosis of cardiac involvement in patients with AFD, considering the focal FDG uptake pattern as presence of active cardiac inflammation. In their cohort, 6 out of 13 patients showed focal LGE indicating myocardial fibrosis, with four of these subjects having simultaneous positive short inversion time inversion recovery (STIR) MR sequences, and focal FDG uptake on the corresponding PET images. Conversely, the patients with positive LGE and negative STIR sequences did not exhibit focal FDG uptake. Therefore, PET/MR imaging may be feasible to distinguish mature fibrosis from fibrosis associated to active inflammation. In a subsequent work, Nappi et al. [57] investigated the role of serial hybrid cardiac 18F-FDG PET/MR in 13 patients with AFD. They observed that between the 8 ERT naïve patients without fibrosis at baseline 18F-FDG PET/MR, those 2 with evidence of focal 18F-FDG uptake after starting ERT showed normal 18F-FDG PET/MR findings at follow-up imaging. These results suggest that AFD course might have been stabilized by treatment before the onset of irreversible cardiac damage. Similarly, in the remaining 6 subjects with normal 18F-FDG PET/MR findings at baseline, no signs of cardiac involvement were observed at follow-up, during ERT. Thus, 18F-FDG PET/MR may be used to follow-up cardiac involvement in AFD at early stage and to monitor disease progression. Imbriaco et al. [58] demonstrated how the hybrid approach can help to personalize the therapeutic pathway for each case, suggesting a potential relationship between progressive accumulation of myocyte sphingolipids and inflammation in the analysis of 20 female AFD patients with 18F-FDG PET/MR imaging. The Authors showed that the coefficient of variation (COV) was higher in patients with focal 18F-FDG uptake compared to those without that (*p* < 0.001). Interestingly, patients with COV > 0.17 had higher T1 values of lateral segments of the mid ventricular wall compared to those with COV ≤ 0.17 (*p* < 0.05). Spinelli et al. [59] demonstrated the relationship between impaired LV longitudinal function with echocardiography and myocardial metabolic abnormalities detected by hybrid PET-MR imaging in the early stage of AFD-related cardiomyopathy in 24 females with genetically proven AFD who had no cardiac symptoms and ERT naïve. An overview of the main studies using hybrid PET/CMR is presented in Table 4.

## 7. Conclusions

Although AFD is a rare cardiac disease, it should be considered among differential diagnoses of those clinical entities presenting with LV hypertrophy. An early diagnosis before organ damage is essential for an optimal management of the patient, allowing to prevent major complications and possibly slowing down the progression of the disease with early therapy initiation. CMR represents the predominant noninvasive imaging modality for the assessment of cardiac involvement in the AFD, being able to comprehensively assess regional anatomy, ventricular function as well as to provide tissue characterization. The most advanced CMR techniques, such as myocardial strain, T1 and T2 mapping, perfusion, and hybrid imaging, have been proved as effective biomarkers either for diagnostic or prognostic purposes.

## Figures and Tables

**Table 1:** Main studies using strain technique in AFD patients.

Author	Year	Aim	Design	Sample	Outcome
Vijapurapu [24]	2018	Relation between early mechanical dysfunction and sphingolipid deposition	Observational, multicentre	221 AFD; 77 controls	GLS correlates with increased LVMi, storage, and ECG abnormalities
Halfmann [25]	2022	Potential of CMR parameters of LA function and strain to detect early stages of disease	Retrospective, single-center	58 AFD; 58 controls	LA reservoir strain showed early impairment and correlated with disease severity

GLS: global longitudinal strain; LVMi: Indexed LV mass; LA: left atrium.

**Table 2:** Main studies using T1 mapping technique in AFD patients.

Author	Year	Aim	Design	Sample	Outcome
Ponsiglione [31]	2022	Weighted mean native T1 values and standardized mean differences determinations	Meta-analysis	14 articles; 982 subjects	Reduction of native T1 values in AFD compared to controls. T1 shortening in AFD influenced by gender and LVH
Camporeale [34]	2019	Functional correlations of T1 values and T1 prognostic value	Prosospective, single-center	44 AFD; 22 controls	In pre-LVH AFD, low T1 values correlate with early ECG and morphological cardiac changes and worsening of systemic disease manifestations
Pica [35]	2014	Electrocardiographic and mechanical correlations of T1 values	Prosospective, single-center	63 AFD; 63 controls	Low T1 values were associated with early diastolic and systolic changes as measured by echocardiography
Thomson [36]	2013	Evaluation of quantitative T1 mapping as a disease-specific imaging biomarker	Multicenter	31 AFD; 23 controls; 21 CR/H subjects	T1 values are the most sensitive and specific CMR parameter for AFD irrespective of sex and LV morphology and function

LVH: left ventricular hypertrophy; CR/H: concentric remodeling or hypertrophy.

**Table 3:** Main studies using T2 mapping technique in AFD patients.

Author	Year	Aim	Design	Sample	Outcome
Augusto [46]	2020	Correlation between chronic edema, myocyte damage and electrical, mechanical, and pathological alterations	Prospective, multicentre	186 AFD; 59 controls; 58 other cardiac diseases	T2 values were higher in AFD patients with LGE. High inferolateral wall T2 was linked to ECG abnormalities
Nordin [47]	2019	Effect of ERT on myocardial storage, inflammation, and hypertrophy	Prospective, multicentre	56 AFD	Over 1 year, patients with advanced disease had increased T2 in areas with LGE and increased troponin.

LGE: late gadolinium enhancement; ERT: enzyme replacement therapy.

**Table 4:** Main studies using hybrid PET/MR imaging in AFD patients.

Author	Year	Aim	Design	Sample	Outcome
Nappi [56]	2015	Assessment of early car-diac involvement by PET/MR imaging	Prospective, single-center	13 AFD	Areas with LGE and positive STIR had focal FDG uptake indicating inflammation
Imbriaco [58]	2019	Effect of ERT on myocardial storage, inflammation, and hypertrophy	Prospective, single-center	20 AFD; 7 controls	Patients with COV >0.17 had higher T1 values in the lateral LV wall compared to those with COV ≤0.17

LGE: late gadolinium enhancement; STIR: short inversion time inversion recovery; FDG: fluorodeoxyglucose; COV: coefficient of variation; LV, left ventricle.

## Data Availability

Data is available on request to the author.
